# Review on Long Non-Coding RNAs as Biomarkers and Potentially Therapeutic Targets for Bacterial Infections

**DOI:** 10.3390/cimb46070449

**Published:** 2024-07-17

**Authors:** Liqin Shi, Xueya Han, Fang Liu, Jinzhao Long, Yuefei Jin, Shuaiyin Chen, Guangcai Duan, Haiyan Yang

**Affiliations:** Department of Epidemiology, School of Public Health, Zhengzhou University, Zhengzhou 450001, China; shilq@gs.zzu.edu.cn (L.S.); snowhan@gs.zzu.edu.cn (X.H.); fliu@zzu.edu.cn (F.L.); ljz069@zzu.edu.cn (J.L.); jyf201907@zzu.edu.cn (Y.J.); sychen@zzu.edu.cn (S.C.); gcduan@zzu.edu.cn (G.D.)

**Keywords:** long non-coding RNAs, bacterial infections, therapeutic targets, biomarkers, regulation pathways

## Abstract

The confrontation between humans and bacteria is ongoing, with strategies for combating bacterial infections continually evolving. With the advancement of RNA sequencing technology, non-coding RNAs (ncRNAs) associated with bacterial infections have garnered significant attention. Recently, long ncRNAs (lncRNAs) have been identified as regulators of sterile inflammatory responses and cellular defense against live bacterial pathogens. They are involved in regulating host antimicrobial immunity in both the nucleus and cytoplasm. Increasing evidence indicates that lncRNAs are critical for the intricate interactions between host and pathogen during bacterial infections. This paper emphatically elaborates on the potential applications of lncRNAs in clinical hallmarks, cellular damage, immunity, virulence, and drug resistance in bacterial infections in greater detail. Additionally, we discuss the challenges and limitations of studying lncRNAs in the context of bacterial infections and highlight clear directions for this promising field.

## 1. Introduction

Bacteria are omnipresent, but only a small proportion of them can cause infections and diseases in the hosts. Bacterial infections are acute systemic infections caused by pathogens or opportunistic pathogens that invade the bloodstream to grow, multiply, and produce metabolites such as toxins [1]. It is estimated that over 7 million deaths worldwide are related to bacterial infections. While antibiotics have revolutionized the treatment of bacterial infections, their misuse has led to the widespread antibiotic resistance in many bacteria. Antibiotic resistance is a rapidly growing global issue with potentially devastating results for modern health systems, often posing greater challenges than infectious diseases caused by viruses and parasites [2,3]. Infections caused by drug-resistant bacteria result in higher morbidity and mortality, complicating treatment efforts. Meanwhile, the lack of new antibiotics has led to the development of alternative treatment strategies to control bacterial infections [4,5,6,7]. However, addressing bacterial infections remains challenging, especially when exploring treatments with epidemic potential, which can result in significant economic losses and medical burden. From the clinical perspective, early identification and diagnosis of microorganisms causing infections, the implementation of targeted prevention and control measures, and effective anti-infective treatment strategies are crucial in combating antibiotic resistance in healthcare. Furthermore, improving antibiotic usage is essential to reduce the disease burden caused by common bacterial infections.

With the advent of high-throughput RNA sequencing (RNA-Seq) technologies, attention has been focused on RNAs that regulate life processes and can be targeted for therapies [8]. Long non-coding RNAs (lncRNAs) are a class of non-coding RNA (ncRNA) molecules longer than 200 nucleotides, lacking open reading frames (ORFs) and without protein coding functions. LncRNAs can regulate a variety of biological pathways at epigenetic and post-transcriptional levels, and are closely related to the processes of human cancer development, invasion, and metastasis [9]. LncRNAs, as important regulators of cellular life activities, are widely involved in the regulation of biological processes such as cell proliferation, differentiation, cycling, and apoptosis. Although their proposed functional scope remains controversial, increasing research evidence suggests that lncRNAs play regulatory roles in the activation of immune signaling cascades, differentiation, and development. For example, they play a key role in a variety of cellular processes such as embryonic development, stem cell pluripotency, neuronal, and cardiac differentiation [10,11]. Notably, they are also associated with invasion and metastasis of malignant tumors such as cervical carcinogenesis, lung, gastric, and breast cancers [12,13].

Additionally, the expression levels of lncRNAs involved in the process of bacterial infections are generally lower than those protein-coding genes, and they exhibit more tissue-specific expression patterns [14]. Studies have shown that drugs can regulate the expression of lncRNAs in vertebrates and invertebrates, suggesting that targeting lncRNAs may offer novel therapies for human diseases [15]. So far, the use of lncRNAs as diagnostic markers and therapeutic agents seems promising since lncRNAs tend to be specifically expressed compared to microRNAs (miRNAs), which are master-regulators in several tissues and often target multiple mRNAs. Enhancing cellular antimicrobial mechanisms, such as autophagy or directly reducing inflammation by influencing the expression of lncRNAs, is a promising approach, especially in the treatment of multi-drug-resistant infections [16]. Evidence indicates that lncRNAs play a key regulatory role in global regulation during bacterial infections [14,16]. Speculating on lncRNAs’ functions based on their processes and locations may help to predict potential mechanisms and identify possible targets for discovering gene regulatory functions during infections. On the basis of the implications in bacteria–host interactions, lncRNAs have been suggested as clinical markers and targets for therapies to prevent or reduce the development of antibiotic resistance in the context of bacterial infections [17].

In this paper, we describe the biogenesis and properties of lncRNAs and focus on their regulatory mechanisms, emphasizing the potential applications of the hallmarks and possible therapeutic targets. This provides new insights into the diagnosis and treatment of bacterial infections. We also further discuss the considerations for the clinical application of lncRNAs in bacterial infections and elucidate its feasibility.

## 2. Biogenesis, Location, and Molecular Regulation of lncRNAs

In contrast to mRNAs, lncRNAs are notably characterized by transcripts longer than 200 nucleotides that do not encode proteins. The biogenesis of lncRNAs is both cell type-specific and stage-specific, involving different mechanisms [18,19]. According to their biogenesis and mechanism of action, lncRNAs can be subdivided into categories such as sense lncRNA, anti-sense lncRNA, intronic lncRNA, enhancer lncRNA, promoter lncRNA, intergenic lncRNA (lincRNA), bidirectional lncRNA, cis-lncRNA, and trans-lncRNA [20]. They are mostly transcribed by RNA polymerase II (Pol II) [9,21], featuring a 5′-cap m7 guanosine and 3′-end poly(A) tail. LncRNAs undergo inefficient splicing and are often retained in the nucleus. Single- or multiple-exon lncRNAs are exported to the cytoplasm via nuclear RNA export factor 1 (NXF1) [22]. Additionally, lncRNAs are more tissue-specific, contain fewer exons, are expressed in lower abundance [23,24], and evolve more rapidly than mRNAs [25].

Due to the rapid evolution of lncRNAs, the processing and localization of lncRNAs have not previously received sufficient attention [26]. For example, the proportion of conserved lncRNAs localized in the cytoplasm of human embryonic stem cells (hESCs) is significantly higher than that in mouse embryonic stem cells (mESCs). Additionally, *FOXD3* antisense transcript 1 (*FAST*) is a positionally conserved lncRNA that exhibits specific expression in both hESCs and mESCs [26]. Many lncRNAs localize on chromatin to interact with proteins, either promoting or inhibiting their binding and activity at targeted DNA regions [24]. LncRNAs located in the nucleus regulate gene transcription and influence mRNAs splicing, stabilization, and translation [27]. Some lncRNAs are also located in membraneless substructures within the nucleus, such as nuclear speckle [28] and nuclear stress bodies [29], and are involved in regulating their assemblies and functions. These suggest that lncRNAs have a complex spatiotemporal regulation in cells, which is related to the localization signals encoded by the sequence. In addition, compared with other tissue sites, lncRNAs are expressed in higher abundance in brain tissue. And studies have shown that lncRNAs play an important role in regulating the development of the nervous system, and are closely related to the occurrence and development of neuropsychiatric diseases such as Parkinson’s disease, Alzheimer’s disease, schizophrenia, and so on [30,31,32]. With the continuous development of sequencing and molecular biology technology, the biological mechanism of lncRNAs in neuropsychiatric diseases is expected to become a hot spot for future research.

**Figure 1:**
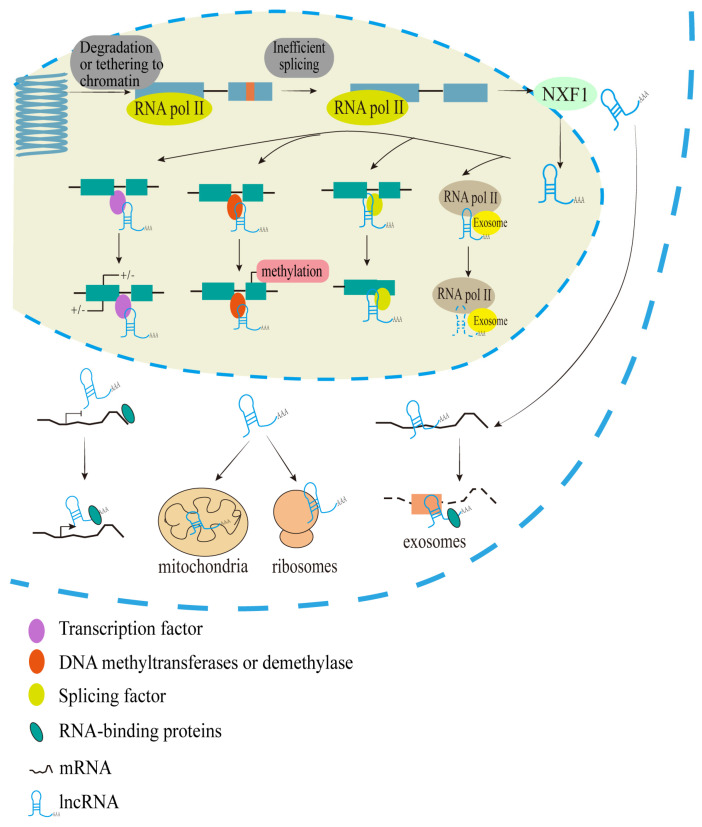
Schematic representation of biogenesis, location, and molecular regulation of lncRNAs. Mechanisms of lncRNAs nuclear retention: A portion of lncRNAs are transcribed by phosphorylation-dysregulated Pol II, resulting in the temporal accumulation of lncRNAs on chromatin, followed by their rapid degradation by RNA exosome. The internal splicing signal of lncRNAs is weak, and the distance between the 3′ splice site and the branch point is long, which is inefficient. Repeat elements may also play a role in driving lncRNAs’ nuclear retention. Mechanisms of splicing and exporting to the cytoplasm: LncRNAs may undergo specific sorting processes, be assigned to specific organelles (such as ribosomes, mitochondria, or exosomes), or be distributed in the cytoplasm and bound to different RNA-binding proteins (RBPs).

Currently, it has been shown that lncRNAs regulate various cellular functions by acting as decoys, scaffolds, guides, sponges, or enhancers at the transcriptional, post-transcriptional, translational, or epigenetic levels [24,33]. LncRNAs are involved in the life process at multiple molecular levels, playing regulatory roles in the nucleus and cytoplasm and interacting with proteins such as chromatin modifiers to regulate innate immunity [34]. Genome-wide detection of RNA–chromatin associations has revealed that lncRNAs can regulate chromatin structure and gene expression through complex interactions [35,36]. Active enhancers can be transcribed into enhancer-associated lncRNAs, which work with chromatin-regulating proteins to influence chromatin structure and topology [37]. So far, most lncRNAs exert their regulatory roles by modulating the transcription of target genomic loci, either in cis (adjacent genes) or in trans (distantly located genes) [38]. Furthermore, the expression patterns of lncRNAs are often correlated with those of mRNAs, both in cis and in trans, suggesting that certain lncRNAs may be co-regulated, sharing similar expression patterns with differentially expressed mRNAs networks [39,40]. Their ability to interact with proteins and nucleic acids allows them to exert post-transcriptional regulation in addition to their roles in chromatin and transcriptional regulation [41,42,43]. LncRNAs can also be processed into small RNAs as a precursor substance and influence the processing of other transcripts by regulating their cleavage into small RNAs or by altering their pre-mRNA splicing patterns [44]. The characterization of the mechanisms involved in the biogenesis, location, and regulation of lncRNAs is illustrated in Figure 1.

## 3. LncRNAs as Biomarkers for the Occurrence and Development of Bacterial Invasion

Pathogen-associated molecular patterns like lipopolysaccharides (LPS) and lipoteichoic acid (LTA) bind to toll-like receptors (TLRs), activating the nuclear factor-κB (NF-κB) transcription factor complex. This activation leads to alterations in lncRNAs [39]. The expression profiles of lncRNAs vary under different conditions and are considered as potential biomarkers for host resistance to infection and immune response modulation [45,46]. On the one hand, the expression profiles of lncRNAs differ among various bacterial infections. For example, infections with *Escherichia coli* (*E. coli*) or *Staphylococcus aureus* (*S. aureus*) specifically regulate lncRNAs’ expressions in a distinct manner [47]. Conversely, the expression profiles of host lncRNAs could also be induced to alter significantly in case of bacterial infections [48,49]. Both in vivo and in vitro studies have shown that the expression patterns of lncRNAs in the groups treated with activated bacteria were notably different from those in control groups [47]. These findings indicate the potential of lncRNAs as clinical markers in the context of bacterial infections.

*S. aureus* is a life-threatening human pathogen. The bacterium expresses alpha-toxin, whose production is controlled by two lncRNAs, SSR42 and RNAIII, regulating cytolytic toxins at the transcriptional and translational levels, respectively [50]. An in vivo and in vitro cross-validation study of homologous lncRNAs identified PRANCR and TNK2-AS1 as stable markers of *S. aureus* mastitis [51]. In case of *Helicobacter pylori* (*H. pylori*) infections, the upregulation of lnc-SGK1 and downregulation of lnc-G protein subunit α transducin 1 (GNAT1) have been linked to human gastric cancer, suggesting their potential as diagnostic indicators [52,53]. Studies on *Mycobacterium tuberculosis* (Mtb) have reported that the expressions of lnc-TGS1-1 and lnc-AC145676.2.1-6 were downregulated in tuberculosis (TB) patients [54], and that PCED1B-AS1, lncRNA XLOC_012582, and ENST00000427151 could be used as early diagnostic biomarkers for Mtb infections [55,56]. Several studies have also investigated the use of lncRNAs as biomarkers for active and latent TB [57,58]. For instance, Fang et al. and Hu et al. identified that differentially expressed lncRNAs, including NONHSAT101518.2 and ENST00000497872, were downregulated in the plasma or blood samples of active TB patients, indicating their promise as clinical diagnostic biomarkers [59,60].

Furthermore, lncRNA maternally expressed gene 3 (MEG3) was downregulated in cells infected with *Mycobacterium bovis* Bacillus Calmette–Guerin (*M. bovis* BCG), but this effect was reversed in *Mycobacterium smegmatis* (*M. smegmatis*) [12]. Nuclear enriched abundant transcript 1 (NEAT1_2) and macrophage interferon regulatory lncRNA 1 (MaIL1) have been reported to be significantly upregulated in cells infected with *Salmonella Typhimurium* and *Legionella pneumophila*, respectively [61,62]. Conversely, lincRNA-EPS and Gm28309 were downregulated during *Listeria monocytogenes* (*L. monocytogenes*) and *Brucella abortus* infections, respectively [63,64]. Transcriptome analysis of epithelial cells from cystic fibrosis patients infected with *Pseudomonas aeruginosa* showed that MEG3, BLACAT1, and MEG9 were significantly downregulated [65]. Moreover, during *Chlamydia trachomatis* infections, FGD5-AS1 was involved in the pathogenic mechanism by regulating the Wnt/β-Catenin pathway [66]. Table 1 summarizes the specific differentially expressed lncRNAs associated with different pathogens [52,61,67,68,69,70,71,72]. These findings suggest that lncRNAs could serve as biomarkers in the systematic early diagnosis, disease progression, and assessment of prognostic effects in bacterial infections.

## 4. LncRNAs as Probable Therapeutic Targets for Bacterial Infections

The complex regulatory mechanisms and low endogenous expression of lncRNAs in specific tissues suggest their potential for targeted therapy in the context of bacterial infections. Currently, there is a growing trend toward RNA therapy, where drugs or antisense oligonucleotides (ASO) target specific RNA sequences or structures to treat diseases [83]. LncRNAs usually interfere with biological processes by affecting the expression of neighboring genes, which also indicates that targeting these gene loci can influence downstream processes for therapeutic purposes. Additionally, altered lncRNAs are associated with the onset and progression of diseases. Due to their tissue specificity, lncRNAs have the ability to target and regulate the gene expression in specific cellular tissues, thereby regulating developmental states and modifying biological processes [84,85]. Despite the enormous potential of lncRNAs-targeted therapies, challenges such as immunogenicity and drug delivery limit their clinical translations. In the following sections, we will explore the potential applications of lncRNAs in pathogenic regulation, cellular damage, especially autophagy and apoptosis, inflammatory processes, immune systems, and drug resistance in bacterial infections.

### 4.1. Targeted Regulation of Pathogenicity by lncRNAs in Bacterial Infections

**Figure 2:**
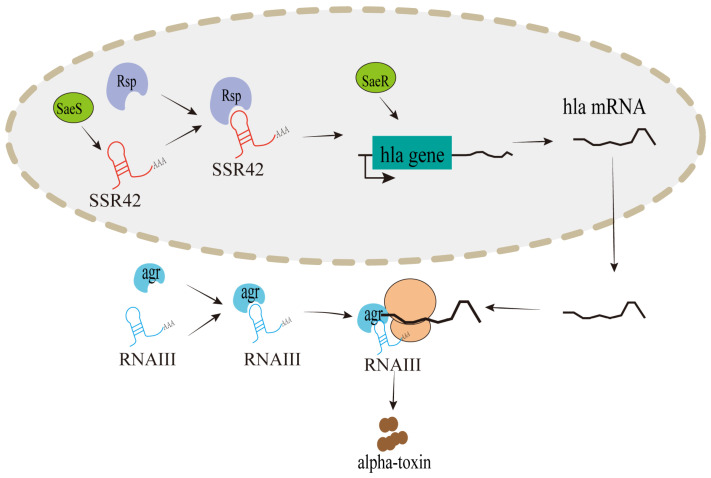
Mechanisms regulation of bacterial pathogenicity by lncRNAs in *Staphylococcus aureus*. SSR42 and RNAIII control the production of cytolytic toxin through virulence regulators at the transcriptional and translational levels, respectively.

Pathogenic bacteria can produce endotoxin, exotoxin, and invasive enzymes, which are the main causes contributing to their pathogenicity. Furthermore, they have evolved numerous survival strategies against the host’s antibacterial systems, primarily through those encoding virulence determinants. These strategies allow pathogens to adapt their metabolic needs and express virulence factors at different stages of infections in a coordinated manner [86]. In particular, lncRNAs play major roles in bacterial gene and virulence regulation [87]. The severity of sepsis caused by *S. aureus* is associated with *staphylococcal* toxins that possess super-antigenic properties, such as toxic shock syndrome toxin-1 (TSST-1) encoded by the *tst* gene [88]. As reported by Safarpour-Dehkordi et al., the expression of LINC00847 was significantly increased in the human renal cell adenocarcinoma (ACHN) transfected with a *tst* recombinant vector compared to those with an empty vector group, and apoptosis and necrosis were also increased [89]. Additionally, two lncRNAs derived from *S. aureus*, SSR42 and RNAIII, regulate the production of cytolytic toxin through the alpha-toxin gene (*hla*) at the transcriptional and translational levels, respectively (Figure 2). As a virulence regulator, the repressor of surface proteins (Rsp) is controlled by SSR42 in the production of the SaeRS-dependent *S. aureus* toxin, which regulates the expression of the *hla* gene in response to antibiotic concentrations [50]. Meanwhile, RNAIII, which blinds the accessory gene regulator (agr), is another major virulence regulator involved in the process of alpha-toxin at the translational level [90].

In summary, lncRNAs are involved in bacterial pathogenicity by regulating virulence genes through specific pathogenic mechanisms. Targeting lncRNAs specifically for pathogenic microorganisms appears to be a promising direction for deciphering the pathogenesis of bacteria.

### 4.2. Targeted Regulation in Cell Lesions Induced by Bacterial Stimulation

After infections with *E. coli* and *S. aureus*, a series of cellular damages occur, primarily manifested as increased apoptosis, stalled cell cycle progression (particularly in the G0/G1 phase), reduced cell viability, and elevated ROS levels [47]. The major function of lncRNAs is to timely regulate the fundamental cellular pathways. At the gene regulation level, the expression of lncRNAs is significantly correlated with these processes. By targeting these cascades, the target genes of lncRNAs are enriched in cellular damage-related pathways, or act as sponges for mRNAs to influence these processes [47]. For example, in response to *S. aureus* stimulation, lnc-AFTR was markedly downregulated and regulated cell damage [91]. Mechanistically, lnc-AFTR reduces cellular damage and inflammation caused by *S. aureus* by directly interacting with FAS mRNA, impeding its translation process without degrading the mRNA. Other lncRNAs, such as SORS1, CD244, and IFNG-AS1, influence autophagy by regulating the transcription of intranuclear genes that interfere with interferon gamma (IFN-γ) and tumor necrosis factor alpha (TNF-α).

Autophagy and apoptosis are common responses to cellular damage, aiming at removing damaged organelles or bacteria. MiRNA-1 indirectly stabilizes the signal transducer and activator of transcription 1 (STAT1) messenger RNA by degrading cytoplasmic lncRNA SROS1, thereby promoting the IFN γ-mediated clearance of *L. monocytogenes* from macrophages [71]. CD244 interacts with the chromatin-modifying enzyme enhancer of zeste homolog 2 (EZH2), which catalyzes H3K27me3 on the IFN-γ and TNF-α promoters, thereby inhibiting their production [68]. The transcription of lncRNA IFNG-AS1 is regulated by STAT4 and T-box transcription factor (T-bet). Additionally, its binding with WD repeat domain 5 (WDR5) stimulates the formation of histone H3 lysine 4 (H3K4) methylation on the IFNG gene, influencing its expression [82]. Furthermore, lincRNA-Cox2 induces and regulates different categories of immune genes, and its expression is induced in an NF-κB-dependent manner [92].

MEG3 might play different roles in the response of different mycobacterial species. Studies have shown dysregulation of MEG3 expression in THP1-derived macrophages infected with *M. bovis* BCG and *M. smegmatis* [70,81]. The mammalian target of the rapamycin (mTOR) signaling pathway acts a pivotal role in autophagy regulation [93]. By linking MEG3 to the mTOR pathway, MEG3 was continuously downregulated in infected macrophages due to IFN-γ-induced autophagy, leading to a significant reduction in intracellular BCG levels [81]. Following Mtb infections, PCED1B-AS1 expression was downregulated as a miR-155 sponge, inhibiting macrophage apoptosis and inducing autophagy [94]. LincRNA-EPS was also downregulated in monocytes from TB patients in in vivo/vitro experiments via the JNK/MAPK signaling pathway, resulting in the inhibition of apoptosis and increased autophagy [80]. Further characterization of these regulatory networks may reveal new drug targets.

**Figure 3:**
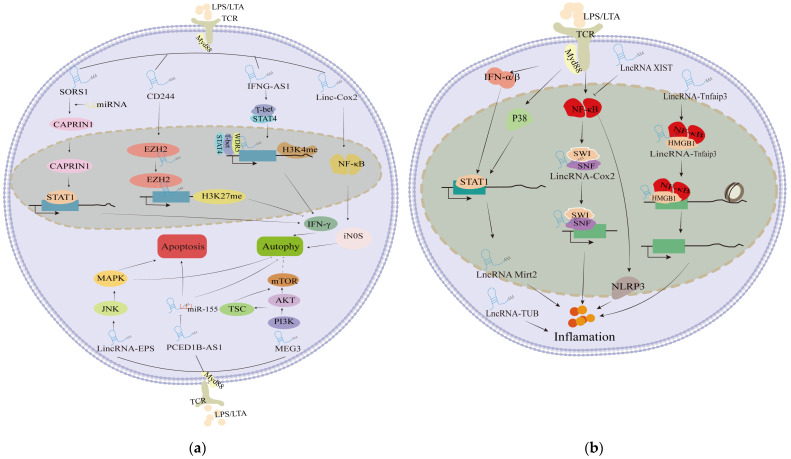
Regulation of lncRNAs in autophagy, apoptosis response, and inflammation of cells to bacterial invasion. (**a**) Regulation of lncRNAs in autophagy and apoptosis response. IFN-γ production is regulated by lncRNAs such as SORS1, CD244, or IFNG-AS1. iNOS production is positively regulated by linc-Cox2. LincRNA-EPS, PCED1B-AS1, and MEG3, which inhibit autophagy, are also dysregulated upon bacterial infections. (**b**) Regulation of lncRNAs in inflammatory pathway. T-cell receptors (TCR) sense extracellular LPS/LTA and activate NF-κB or type I IFN production through the myeloid differentiation primary response protein 88 (MyD88) pathway.

These studies highlight the roles of lncRNAs in the autophagy and apoptosis response of cells to bacterial invasion (Figure 3a). In brief, lncRNAs can regulate pathways related to cellular damage, thereby regulating processes under bacterial stimulation through downregulation or upregulation. Similarly, inducing the construction of overexpression or knockout of the corresponding lncRNAs can achieve the desired regulatory effect. It is worth mentioning that lncRNAs are often treated with oligonucleotide therapies, and novel delivery platforms have expanded the applicability of lncRNA-targeted drugs. For the therapy of lncRNAs, targeting diseased tissues with oligonucleotides remains challenging due to unknown immune responses and issues including tolerability.

### 4.3. Targeted Mediation of lncRNAs Inflammation Process Induced by Bacterial Infections

During bacterial infections, immune cells near the affected area are activated and release cytokines to regulate the process of inflammation. These cytokines act as signals, summoning immune cells elsewhere to join the fight and promptly assess the inflammatory process. The expression of lncRNAs is significantly correlated with the inflammatory process, with NF-κB being considered a crucial signaling pathway (Figure 3b). For example, lncRNA XIST regulates the expression of the NLRP3 inflammasome by mediating the NF-κB pathway, forming a negative feedback loop to modulate the inflammatory process. Silencing of XIST enhances the expression of pro-inflammatory cytokines induced by *E. coli* or *S. aureus* [95]. Similarly, another lncRNA Mirt2, upregulated in LPS-induced macrophages, was described in more detail in a time- and dose-dependent manner. Mirt2 inhibited TNF receptor-associated factor 6 (TRAF6) oligomerization and ubiquitination by activating the p38 and IFN-α/β pathways, effectively alleviating inflammation [96]. However, the exact human homolog of Mirt2 has not been determined. Specific lncRNAs also play a role in modulating multiple inflammatory factors simultaneously. For example, knockdown of lncRNA-TUB promotes interleukin (IL) -1β expressions induced by *E. coli*, while TNF-α and IL-6 have the opposite effect, leading to an imbalanced inflammatory stress state [39].

LincRNAs may serve as key mediators in regulating NF-κB gene transcription and participating in the pathogenesis of bacterial infections, making them targets for therapeutic intervention. For example, following TLR4 stimulation triggered by LPS, lincRNA-Cox2 assembles into the switch/sucrose nonfermentable (SWI-SNF) complex in macrophages, facilitating the assembly of NF-κB subunits into the SWI-SNF complex, thereby activating late-primary inflammatory response genes [97]. LincRNA-Tnfaip3 is also upregulated in response to LPS and potentially acts as a scaffold molecule to stabilize the high mobility group box 1 (HMGB1)/NF-κB complex and modulate associated histone modifications, promoting the transcription of NF-κB-controlled inflammatory response genes [98]. Knockdown of lincRNA-Tnfaip3 results in the downregulation of inflammatory gene expression, further validating these findings [98].

In summary, different lncRNAs target and regulate the expression of inflammatory genes at various genetic levels, influencing key pathways and ultimately producing inflammatory factors. Moreover, the specific regulatory feedback of lncRNAs varies across tissues and stages of disease development.

### 4.4. Targeted Modulation of lncRNAs in the Immune System of the Antibacterial Parade

**Figure 4:**
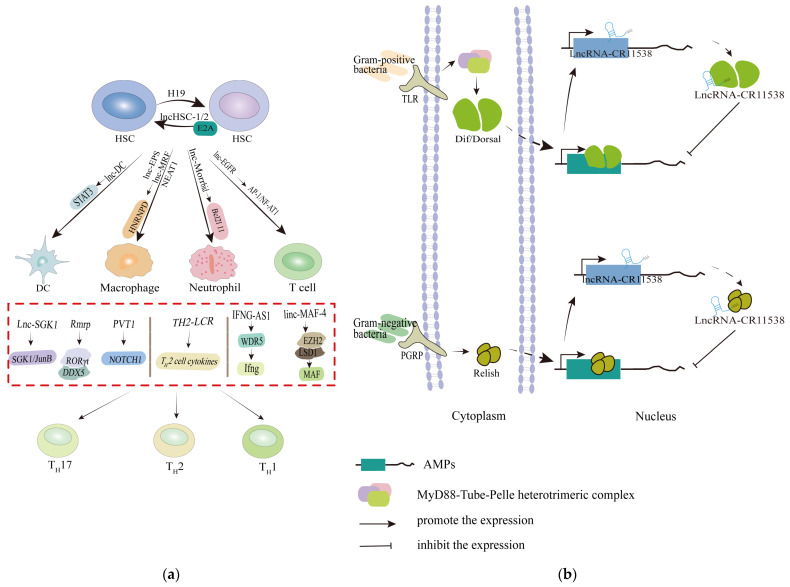
Targeted regulation of lncRNAs in the immune system. (**a**) Differentiation and function lineage determination of immune cells by lncRNAs. LncHSC is involved in E2A binding at target sites and affects HSC self-renewal. Lnc-DC and lncRNA Morribid regulate immunity through STAT3 phosphorylation and the pro-apoptotic factor Bcl2l11, respectively. Lnc-EGFR activates the downstream AP-1/NF-AT1 axis to promote T-regulatory cell differentiation. In addition, lncRNA SGK1, Rmrp, PVT1, IFNG-AS1, and MAF-4 induce or inhibit T_H_1, T_H_2, and T_H_17 differentiation by regulating various pathways. (**b**) The mechanism of lncRNAs restoring Toll immune homeostasis in *Drosophila*. Antimicrobial peptide genes are regulated by a balance between two signaling pathways: the Toll pathway, which is activated primarily by Gram-positive bacteria, and the Imd (immune deficiency) pathway, which is activated primarily by Gram-negative bacteria. The Toll pathway, which recruits intracellular Death domain-containing proteins, MyD88, Tube, and Pelle, early promotes Dil/Dorsal entry into the nucleus and enhances AMP transcriptions. Later redundant AMPs increase the expression of lncRNA-CR11538, which serves as a decoy to downregulate the expression of AMPs to restore immune homeostasis. Nuclear transposition of Relish results in the massive activation of AMPs after activation of the Imd signaling pathway by Gram-negative bacteria [108]. With increased AMPs concentration and the elimination of pathogens, Relish activates lncRNA-CR11538 transcription, decoying itself away from the AMPs promoter region. This subsequently suppresses the expression of AMPs and maintains immune homeostasis. HSC: hematopoietic stem cells; DC: dendritic cells; TLR: Toll-like receptor; PGRP: peptidoglycan recognition protein; AMPs: antimicrobial peptides.

The lncRNAs transcriptome of immune cells is highly dynamic, exhibiting major changes related to infection response during development, differentiation, and activation processes. Compared to miRNAs, lncRNAs are highly expressed and distributed in immune organs and cells, playing a crucial role in regulating immune response. In the immune system, the expression of lncRNAs is context-, developmental stage- and cell type-specific, coordinating many aspects of immune function [39]. In particular, adaptive immune system cells, such as T cells, monocytes, macrophages, etc., are commonly used to study lncRNAs in vivo. Antimicrobial immunity relies on the complex interplay between innate and adaptive immune responses. Notably, a considerable number of lncRNAs have the potential to participate in normal and pathological states by regulating the biological processes such as differentiation and function lineage determination of immune cells [20] (Figure 4a). However, the literature on this topic is primarily derived from animal studies, emphasizing the need for validation in human cells.

LncRNAs coordinate the expression of cell-specific genes, regulating the differentiation of precursor cells into mature cells and subsequent activation [20]. Long-term hematopoietic stem cells (LT-HSCs) have high expression of H19, which is immediately downregulated once differentiated into short-term HSCs. H19-deficient LT-HSCs lose the ability to self-renew and instead enter the cell cycle and differentiate into downstream cell types [99]. LncHSC, expressed specifically in HSCs in vitro, could participate in E2A binding at target sites, with knockout leading to increased myeloid differentiation and affected HSC self-renewal [100]. However, the molecular mechanisms remain unclear, and lncRNAs in the development of megakaryocytes are yet to be extensively explored. Dendritic cells (DC) play a central role in the host’s innate immune defense against pathogens. Lnc-DC controls differentiation by promoting STAT3 phosphorylation and nuclear translocation [101]. Its knockdown inhibited DC differentiation from human monocyte and mouse myeloid cells, negatively regulating immune responses [102]. Monocytes/macrophages act as sentinels in the host’s immune defense system. A recent study using a human monocyte engraftment mouse model confirmed that downregulation of lncRNA MRF inhibited monocyte chemotaxis, indicating its potential as a target for mesenchymal stem cell-related immune regulation [103]. NEAT1 has also been identified as a novel lncRNA-type immunomodulator affecting monocyte–macrophage function [61].

Additionally, lncRNAs play a more specific immunoregulatory function in the differentiation program of T cells. For instance, lnc-EGFR stimulated regulatory T-cell differentiation, inducing immune suppression [104]. Lnc-SGK1 was upregulated in *H. pylori* infections, further enhancing the transcription of serum and glucocorticoid-inducible kinase 1 (SGK1), thereby amplifying the SGK1/JunB pathway to induce T_H_2 and T_H_17 differentiation and inhibit T_H_1 differentiation [52]. Overexpressing lncRNA PVT1 might inhibit T_H_17 cell differentiation by downregulating the Notch pathway [105]. LincRNA MAF-4 repressed the expression of the T_H_2 cell transcription factor MAF, promoting T cell differentiation toward the T_H_1 cell lineage. Simultaneously, knockdown of linc-MAF-4 altered T-lymphocyte toward T_H_2 cell lineage differentiation by changing MAF transcription, which alters the function of chromatin modifiers [106]. Furthermore, lncRNA Morrbid controlled apoptosis of highly inflammatory cells such as eosinophils by regulating the expression of the pro-apoptotic factor Bcl2l11, and the Morrbid-AKT axis might be important in other environments and cell types that express this lncRNA [107]. Table 2 provides an overview of lncRNAs for probable therapeutic targets in bacterial diseases.

LncRNAs also play a critical role in maintaining immune homeostasis. For instance, in *Drosophila*, the Toll signaling pathway induces the NF-κB transcription factor Dif/Dorsal to translocate into the nucleus through a series of signaling cascades, thereby activating the expressions of antimicrobial peptides (AMPs) in response to Gram-positive bacteria invasion [108]. Overexpression of lncRNA-CR11538 acts as a decoy in the nucleus, guiding Dif/Dorsal away from the promoters of AMPs. This action inhibits the expression of AMPs and promotes the restoration of immune homeostasis (Figure 4b) [109]. Overall, our current understanding indicates that lncRNAs function in the regulation of immune processes, providing new insights into the study of the immune system. They appear to act as tunable and specifically expressed immune modulators to combat bacterial invasion.

Although many preclinical studies have investigated lncRNAs as therapeutic targets, converting these findings into viable treatments remains challenging. These challenges significantly impeding the conversion of accumulated research into meaningful clinical trials must be emphasized. The substantial corpus of research in the files confirms the therapeutic promise of lncRNAs. Even though no nucleic acid-based therapeutic approach directly targeting lncRNAs has yet reached the clinic, their potential as therapeutic agents is undeniable.

**Table 2:** Summary of lncRNAs for probable therapeutic targets in bacterial diseases.

Pathogen	LncRNA	Regulation	Target Cells	Mechanisms	References
*S. aureus*	SSR42	Up	Encoded by bacteria	Involves in *S. aureus* hemolysis induced by antibiotics	[50]
	LINC00847	Up	Renal cancer cells	Arrests cell cycle and results in activation of apoptosis through regulatory lncRNA and *tst* gene	[89]
	RNAIII	Up	Encoded by bacteria	Regulates staphylococcal alpha-toxin at the translational level through base pairing with HLA mRNA	[90]
	lnc-AFTR	Down	*S. aureus*-induced MAC-T cells and mastitis tissues	Inhibits apoptosis and inflammation by blocking FAS mRNA translation	[91]
*L. monocytogenes*	SROS1	Down	Macrophage	Degraded by upregulated miR-1 to release RBP CAPRIN1, thus improving the stabilization of Stat1 mRNA	[71]
	lincRNA-EPS	Down	Macrophage	Interacts with hnRNPL to repress immune-responsive gene-1 expression	[72]
	lincRNA-Cox2	Up	Macrophage	Regulates the assembly of NF-κB subunits to the SWI/SNF complex, thereby promoting late inflammatory gene transcription	[92,110]
	Morrbid	Up	Eosinophils, Macrophage	Controls apoptosis of highly inflammatory cells such as eosinophils by regulating the expression of the pro-apoptotic factor Bcl2l11	[107]
	AS-IL1α	Up	Macrophage	RNA polymerase II recruitment to the IL-1α promotor	[79]
Mtb	lncRNA CD244	Up	CD8^+^ T-cells	Recruits Ezh2 to mediate the trimethylation of H3K27 in IFN-γ/TNF-α locus	[68]
	NEAT1	Up	Monocyte, Macrophage	Regulates IL-6 expression	[69]
	PCED1B-AS1	Down	Macrophage	As a miR-155 sponge, inhibiting macrophage apoptosis by regulating FOXO3/Rheb	[94]
	lincRNA-Cox2	Up	Macrophage	Regulates the inflammatory response of TB infection macrophages through Stat3 and NF-κB signaling pathways	[97]
	HOTAIR	Up (H37Ra-infected) Down (H37Rv-infected)	Macrophage	Likely regulates the expression of DUSP4 and SATB1 by recruiting EZH2 during infection	[111]
*Salmonella*	NEAT1	Up	HeLa cells	Alters expression of host immune genes	[61]
	IFNG-AS1	Up	Macrophages and murine tissues	Interacts with WDR5 to promote histone methylation at the IFN-γ locus	[82]
*M. smegmatis*	MEG3	Up	Macrophage	Inhibits the expression of TGF-β by forming RNA-DNA triplex structures	[70]
*M. bovis* BCG	MEG3	Down	Macrophage	IFN-γ-induced autophagy	[81]
Pulmonary tuberculosis; *M. bovis* BCG	lincRNA-EPS	Down	Monocyte, Macrophage	Regulation of apoptosis and autophagy	[80]
*E. coli* or *S. aureus*	lncRNA-TUB	Up	MAC-T cells	Regulates the morphology, proliferation, migration, and β-casein secretion of mammary epithelial cells	[39]
	lncRNA-XIST	Up	MAC-T cells	Inhibits *E. coli* or *S. aureus*-induced NF-κB phosphorylation and the production of NLRP3 inflammasome	[95]
*E. coli*	lncRNA-Mirt2	Up	Macrophage	Inhibits the K63-ubiquitination of TRAF6 and thus alleviates inflammatory responses after TLR4 activation and balances macrophage polarization	[96]
LPS stimulation	lincRNA-Tnfaip3	Up	Macrophage	Acts as a coactivator of NF-κB for the transcription of inflammatory genes through modulation of epigenetic chromatin remodeling	[98]
	lncRNA-MRF	Up	Monocyte	Increases monocyte recruitment by upregulating the expression of monocyte chemotactic protein	[103]
*H. pylori*	lnc-SGK1	Up	T cells in human gastric cancer	Enhances SGK1 transcription in cis to induce T_H_2 and T_H_17 differentiation and inhibit T_H_1 differentiation	[52]
	lnc-GNAT1	Down	Gastric cancer cells	Impedes cancer cell migration through Wnt/β-catenin pathway protein expression inhibition	[53]
	ZFAS1	Up	Colorectal cancer tissues	Interacts with CDK1 to be involved in p53-dependent cell cycle control and apoptosis	[77]
*P. aeruginosa*	MEG3-4	Down	Macrophage, Epithelial cells	Bindings to miRNA-138, thereby releasing interleukin-1β to augment inflammatory responses	[112]

Abbreviations: MAC-T: Bovine mammary epithelial cell lines; CDK1: Cyclin-dependent kinase 1.

### 4.5. Could lncRNAs Be Targeted as a Promising Solution to the Current Antibiotic Resistance?

In dealing with bacterial infections, the problem of antibiotic resistance has always been a major clinical focus. To combat bacterial antimicrobial resistance, innovative methods such as phage therapy, CRISPR-Cas9 technology, and host-directed therapy have been investigated in the early stages. Exposure to subinhibitory concentrations of antibiotics can enhance alpha-toxin production and alter disease progression in *S. aureus*, a process that is dependent on lncRNA SSR42 [50]. Several studies have also explored the expression profiles of lncRNAs related to the antagonistic effects of *E. coli* F17 on lamb spleens [113,114]. Additionally, two other studies reported differentially expressed lncRNAs in the immune response to multidrug-resistant tuberculosis (MDR-TB) infections, revealing that some lncRNAs might be involved in regulating host immune response to MDR-TB infections [115,116]. The Kyoto Encyclopedia of Genes and Genomes (KEGG) analysis showed that the differential mRNAs between drug-sensitive tuberculosis (DS-TB) and MDR-TB were mainly enriched in the proteasome and the Notch signaling pathway, potentially providing new clues for investigating potential therapeutic targets for MDR-TB [116]. In addition, lncRNA SNHG3, acting as a “sponge” of miR-139-5p, promoted enzalutamide-induced resistance, which might be an effective metabolism target for anti-prostate cancer therapy [117]. LINC00152 and lncRNA AK022798 regulated drug resistance by targeting the Notch signaling pathway [118].

**Figure 5:**
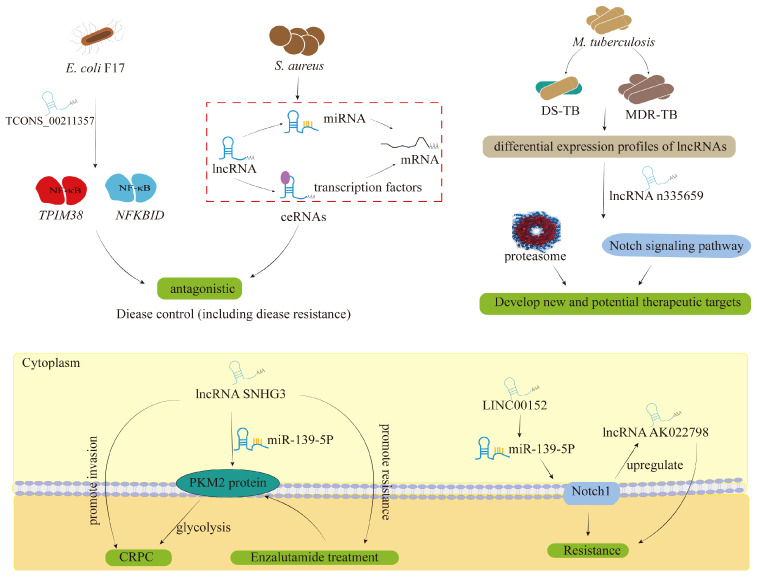
LncRNA TCONS_00211357, which is predicted to target several NF-κB pathway-related genes such as *TRIM38* (NF-κB activator) and *NFKBID* (NF-κB inhibitor), may act as a key regulator of the NF-κB pathway in immune response to *E. coli* F17 infection. Targeting signaling pathways of *E. coli* and *S. aureus* maternal genes have similar functions and provide a basis for screening effective therapeutic agents through lncRNA-miRNA-mRNA interactions networks. The detection of serum lncRNA n335659 may have potential for monitoring the effect of tuberculosis treatment. LncRNA SNHG3 may regulate the expression of PKM2 through binding to miR-139-5P competitively, and thus regulate CRPC development and enzalutamide resistance. *E. coli*: *Escherichia coli*; *S. aureus*: *Staphylococcus aureus*; ceRNAs: competitive endogenous RNAs; lncRNA: long non-coding RNA; miRNA: microRNA; *M. tuberculosis*: *Mycobacterium tuberculosis*; DS-TB: drug-sensitive tuberculosis; MDR-TB: multidrug-resistant tuberculosis; PKM2: pyruvate kinase isozyme typeM2; CRPC: castration-resistant prostate cancer.

It seems feasible that clinical applications of lncRNAs could be used to deal with drug resistance in bacterial infections (Figure 5). However, the resistant mechanisms described above have not been thoroughly investigated, and the approach to combating bacterial resistance remains uncertain. Additionally, research on the molecular mechanisms involving lncRNAs in the resistance process is scarce, with most studies focusing on a few types of cancers. Interestingly, the key risk factors for cancers have been associated with bacterial infections [119]. Research on the mechanism of tumor drug resistance closely related to bacterial infections may provide some clues to bacterial resistance as well.

## 5. Conclusions and Future Outlook

LncRNAs play crucial roles in molecular mechanisms of cellular damage, immunity, virulence, and drug resistance associated with bacterial infections. Their tissue-specific and condition-specific expression patterns indicate that lncRNAs could serve as potential biomarkers and provide a rationale for their clinical targeting. In bacterial infections, many lncRNAs participate in IFN-γ reactivity and autophagy-dependent antimicrobial defense mechanisms. However, interferon- and NF-κB-driven immune responses can become detrimental, leading to life-threatening pathology during infections. This phenomenon reveals the highly complex interactions between host and bacterial pathogens. It is worth mentioning that current studies mainly focus on screening differentially expressed lncRNAs as clinical markers, with less emphasis on exploring the molecular mechanisms of specific lncRNAs in the preclinical stage of bacterial infections [120]. Future preclinical studies in mice to further identify human immunoregulatory lncRNA loci could help to understand their in vivo relevance during bacterial infections.

However, poor sequence conservation across species remains a key obstacle in researching the functional applications of lncRNAs. To overcome this, tools for discovering more structurally conserved homologues need to be developed. One promising strategy is to use humanized mouse models to study the response of lncRNAs to bacterial infections. Moreover, in addition to clinically approved RNA antagonists, advances in the development of small-molecule RNA inhibitors have received widespread attention in recent years. Currently, lncRNAs are mostly used for targeted therapy of drug-resistant cancers, but data on their applications in bacterial treatments are scarce. These are all exploratory directions for future research. Given the widespread use of antibiotics and the emergence of drug-resistant bacteria, studying the targeted therapy and resistant mechanisms of lncRNAs in bacterial infections will provide valuable insights.

## Figures and Tables

**Table 1:** Representative lncRNAs as biomarkers in the different infections of bacteria.

Pathogen	LncRNA	Expression	Role	References
*Brucella* spp.	Gm28309	Down	Shows potential as a biomarker in the disease progression for brucellosis	[64]
	IFNG-AS1	Up	Enhances the expression of INF-γ in the immune response of brucellosis patients, revealing its potential in screening, diagnosis, or treatment of brucellosis	[73]
*C. trachomatis*	FGD5-AS1	Up	Upregulated FGD5-AS1 provides new ideas for screening potential intervention targets for *C. trachomati*	[66]
	lncRNA-IRF1	Up	MIAT and IRF1 are involved in apoptosis resistance in persistent infection	[74]
	MIAT	Up		[74]
	ZEB1-AS1	Up	A persistent *C. trachomatis* infection up-regulated lncRNA that could enhance persistent infection-induced anti-apoptosis	[74]
	ZFAS1	Up	Plays a role in the anti-apoptotic process of *C. trachomatis*	[75]
*E. coli*	LRRC75A	Down	Knockout of LRRC75A-AS1 attenuated the *E. coli*-induced inflammatory responses	[76]
*H. pylori*	lncRNA GNAT1-1	Down	Regulation of invasion and migration of gastric cancer cells	[53]
	ZFAS1	Up	Upregulation of lncRNA ZFAS1 predicts poor prognosis and promotes invasion and metastasis in colorectal cancer	[77]
*L. monocytogenes*	lincRNA-EPS	Down	Downregulation of lincRNA-EPS leads to an increased production of proinflammatory cytokines and iNOS in splenic and liver macrophages and dendritic cells	[63]
	lncRNA-SROS1	Down	Shows potential as biomarkers in the disease progression	[71]
	lincRNA-Cox2	Up		[78]
	AS-IL1α	Up	An important regulator of IL-1α transcription during the innate immune response, and has the potential to be used as a diagnostic marker	[79]
*M. bovis*	lincRNA-Cox2	Up	Shows potential as biomarkers in the disease progression	[78]
	lincRNA-EPS	Down		[80]
	MEG3	Down	Shows potential as a biomarker in the systematic early diagnosis	[81]
*M. smegmatis*	MEG3	Up	As a novel mediator of host cell response during mycobacterial infections	[70]
*M. tuberculosis*	XLOC_012582	Up	These deregulated lncRNAs in Mtb-infected cells can be introduced as promising molecular biomarkers for prognosis and/or diagnosis	[56]
	PCED1B-AS1	Up		[56]
	lncRNA-CD244	Up	A major regulator of CD8 T-cell immune response during in vivo Mtb infection and has the potential to be used as a diagnostic marker	[68]
	NEAT1	Down	Tuberculosis patients had significantly increased the expression of NEAT1, and NEAT1 might serve as a potential indicator for patient prognosis of tuberculosis	[69]
*S. aureus*	TNK2-AS1	Up	TNK2-AS1 could be regarded as stable markers associated with bovine *S. aureus* mastitis	[51]
*S. typhimurium*	NEAT1_2	Up	Shows potential as biomarkers in the systematic early diagnosis	[61]
	IFNG-AS1	Up		[82]

Abbreviations: *C. trachomatis*: *Clamydia trachomatis*; *E. coli*: *Escherichia coli*; *H. pylori*: *Helicobacter pylori*; *L. monocytogenes*: *Listeria monocytogenes*; *M. bovis*: *Mycobacterium bovis*; *M. smegmatis*: *Mycobacterium smegmatis*; *M. tuberculosis*: *Mycobacterium tuberculosis*; *S. aureus*: *Staphylococcus aureus*; *S. typhimurium*: *Salmonella typhimurium*.

## Data Availability

The data that support the findings of this study are included in this article and are available from the corresponding author upon reasonable request.
